# [Corrigendum] Knockdown of Bmi1 inhibits the stemness properties and tumorigenicity of human bladder cancer stem cell-like side population cells

**DOI:** 10.3892/or.2026.9177

**Published:** 2026-08-10

**Authors:** Dingjun Zhu, Xuesi Wan, Hai Huang, Xu Chen, Wu Liang, Fengjin Zhao, Tianxin Lin, Jinli Han, Wenlian Xie

Oncol Rep 31: 727–736, 2014; DOI: 10.3892/or.2013.2919

Following the publication of the above article and an Expression of Concern statement that was issued in light of concerns raised by an interested reader (doi: 10.3892/or.2025.9006) regarding the possible presentation of the same mouse twice in Fig. 7A, which showed how Bmi1 silencing suppresses the tumorigenicity of SP T24 cells *in vivo*, the authors have now responded to the enquiry posed by the Editorial Office. After consulting their original data, the authors have realized that the images of the first and third mouse in Fig. 7A were inadvertently mistakenly used during the manuscript preparation and figure editing stage.

The authors were also asked to offer an explanation for the apparent discrepancy in the tumor sizes, given that the sizes of the tumors *in situ* appeared to be significantly larger than the measured sizes of the associated excised tumors. The authors acknowledged this concern, and attributed it to visual distortion from skin/fur, together with non-standard approximate size recording practices at the time, while also admitting that detailed original measurement data were no longer available.

The revised version of Fig. 7, now showing the correct mice in Fig. 7A, is shown on the next page. Note that the errors made in assembling this figure did not affect the overall results and conclusions reported in the paper. The authors are grateful to the Editor of *Oncology Reports* for granting them the opportunity to publish this corrigendum, and all the authors agree with its publication; furthermore, they apologize to the readership of the journal for any inconvenience caused.

## Figures and Tables

**Figure 7.**
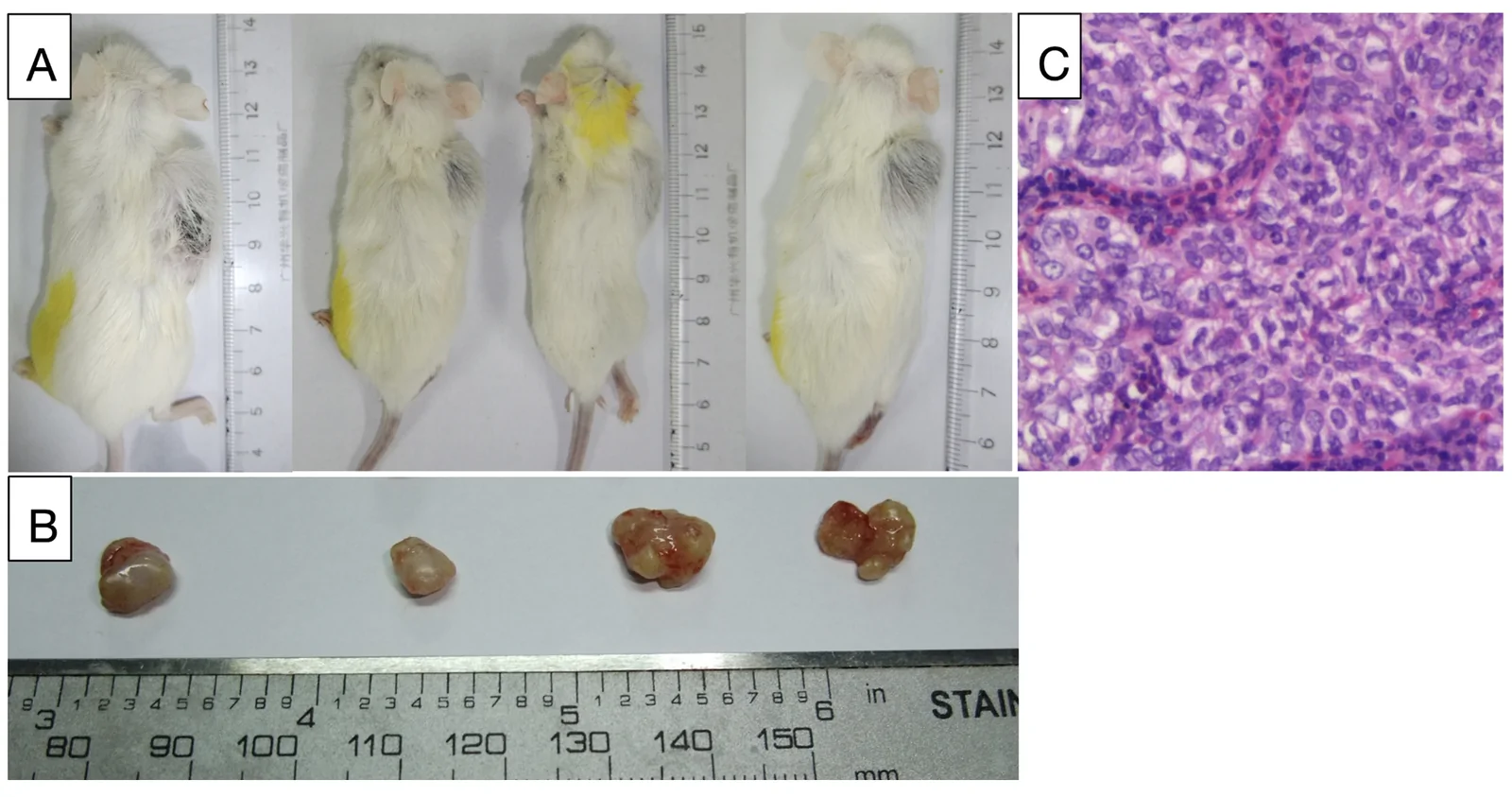
Bmi1 silencing suppresses the tumorigenicity of SP T24 cells *in vivo.* (A) SP-Bmi1-siRNA and SP-scramble-siRNA T24 cells (1x105) were subcutaneously injected into the left and right sides of the back of recipient mice, respectively. The mice were sacrificed 6 weeks later. (B) Sizes of the tumors formed from subcutaneously injected SP-scramble-siRNA T24 cells. (C) Results of hematoxylin and eosin (H&E) staining showed that the tumor formed from subcutaneously injected SP-scramble-siRNA T24 cells was a typical urothelium carcinoma (magnification, x100).

